# Exploring the use of translation technologies to overcome language barriers in mental healthcare: a qualitative cross-country study

**DOI:** 10.1186/s12939-026-02821-2

**Published:** 2026-03-20

**Authors:** Annika Kreienbrinck, Saskia Hanft-Robert, Muhammed-Talha Topçu, Lena Peters, Ovidiu Oltean, Asithandile Nozewu, Houda Al-Kalaf, Andrian Liem, Alina Ioana Forray, Rowan Madzamba, Leslie Swartz, Christine Anthonissen, Ted Sanders, Brian Hall, Barbara Schouten, Christopher Jenks, Răzvan Cherecheș, Mike Mösko

**Affiliations:** 1 Department of Medical Psychology, University Medical Center Hamburg-Eppendorf, Martinistraße 52, Building West 26, 20246 Hamburg, Germany; 2 Department of Applied Human Sciences, Magdeburg-Stendal University of Applied Sciences, Stendal, Germany; 3 Faculty of Political, Administrative and Communication Sciences, Babeș-Bolyai University, Cluj-Napoca, Romania; 4 Department of Community Medicine, Discipline of Public Health and Management, Iuliu Hațieganu University of Medicine and Pharmacy, Cluj-Napoca, Romania; 5 Department of Psychology, Stellenbosch University, Stellenbosch, South Africa; 6 Faculty of Humanities, Institute for Language Sciences, Utrecht University, Utrecht, The Netherlands; 7 Jeffrey Cheah School of Medicine and Health Sciences, Monash University Malaysia, Subang Jaya, Malaysia; 8 Faculty of Psychology, Universitas Sebelas Maret, Surakarta, Indonesia; 9 The Center for Global Health Equity, NYU Shanghai, Shanghai, People’s Republic of China; 10 Faculty of Social and Behavioural Sciences, Amsterdam School of Communication Science (ASCoR), Centre for Urban Mental Health, University of Amsterdam, Amsterdam, The Netherlands; 11 Department of Public Health, Babeș-Bolyai University, Cluj-Napoca, Romania; 12 Division of Health Systems and Public Health, Faculty of Medicine and Health Sciences, Stellenbosch University, Stellenbosch, South Africa

**Keywords:** Translation technologies, Language barriers, Mental healthcare, Multilingualism, Machine translation, Digital health, Qualitative research, Cross-country study, Health communication, Equity in healthcare

## Abstract

**Background:**

Language barriers in healthcare continue to pose major challenges to equitable, high-quality care, particularly in mental health settings where communication is central to diagnosis, therapeutic alliance, and treatment planning. Translation technologies, including machine translation applications, offer potential solutions, but their real-world usability in mental health care remains underexplored. This cross-country qualitative study examined the experiences, attitudes, needs, and suggestions regarding the use of translation technologies to overcome language barriers in mental health care.

**Methods:**

Semi-structured interviews were conducted with 175 participants, including healthcare providers, interpreters, service users, supporters, and stakeholders (e.g., policymakers, community organisation representatives), across five countries: Germany, South Africa, Romania, the Netherlands, and China. The data were analysed thematically using a hybrid deductive-inductive approach, with Bronfenbrenner’s ecological systems model applied as a sensitising framework.

**Results:**

Six overarching themes emerged: (1) perceived effectiveness and limitations of translation tools, (2) cultural, linguistic, and social fit, (3) human aspects of communication and care, (4) ethical and attitudinal dimensions, (5) implementation and improvement pathways, and (6) access barriers. Participants often found the translation tools to be helpful for basic communication but inadequate for complex or emotional interactions central to mental health care. Key concerns included translation accuracy limitations, cultural inappropriateness, reduced empathy and trust, data protection issues, and poor workflow integration. Infrastructure constraints, institutional barriers, and lack of formal guidance hindered effective implementation. Participants emphasised the importance of hybrid models combining technology with human interpreters, tailored training, and clear policy frameworks to address these challenges.

**Conclusion:**

Translation technologies can help bridge language gaps in mental healthcare, particularly in urgent or resource-constrained settings. However, their effective use remains highly context-dependent and constrained by technical, ethical, relational, and infrastructural challenges. Adequate and equitable implementation requires system-level investment, participatory design, and safeguards that protect both the emotional and cultural dimensions of communication, among others. Translation tools should be seen as supplementary, not substitutive, in delivering safe and person-centred mental healthcare.

**Clinical trial registration:**

Not applicable.

**Supplementary Information:**

The online version contains supplementary material available at 10.1186/s12939-026-02821-2.

## Introduction

Language barriers are a common challenge in healthcare worldwide, affecting access to care, the quality of treatment, and service user outcomes [1–3]. Effective verbal communication is crucial in mental healthcare, where symptoms are often complex and not directly observable, making accurate assessment and treatment highly dependent on language [4].

The context for this study encompasses five diverse countries with distinct linguistic landscapes, healthcare infrastructures, and migration histories: Germany, South Africa, Romania, the Netherlands, and China. These countries were selected to capture a range of health system contexts and to distinguish between universal and context-specific challenges, thereby situating language barriers within broader global health inequities. In a study conducted in the Netherlands, nearly 40% of hospital patients who participated and belonged to an ethnic minority group reported having limited communication skills in the dominant language [5]. In South Africa, where there are 12 official national languages, more than 80% of medical consultations take place with language barriers, with only 6% of medical interactions fully or partially conducted in the service user’s native language [6]. In Romania, 13% of migrant study respondents reported limited access to health services due to language barriers [7]. In China, studies highlight existing disparities in healthcare and access to healthcare among different migrant groups [8, 9]. In Germany, approximately 37% of study participants with migration experience self-reported low German proficiency after more than 11 years of residence, and 12% of service users with migration experience in oncological or psychiatric outpatient care reported insufficient language skills to fully participate in consultations [10, 11].

Language discordance can undermine therapeutic alliance, lead to misunderstandings, misdiagnosis, reduced treatment adherence, and early discontinuation of care [2–4]. Yet they represent just one of many intersecting challenges, including structural, socio-economic, and legal barriers, that limit access to care for linguistically diverse populations [12, 13]. Despite widespread recognition of the importance of communication in providing care, addressing language barriers remains a persistent challenge in research, education, and clinical practice. In many countries, the responsibility for overcoming these barriers is placed on migrants, who are expected, implicitly or explicitly, to learn the dominant language, rather than on healthcare systems to adopt more inclusive and system-level approaches [14, 15].

Healthcare providers and service users frequently rely on informal strategies, including informal interpreting by family members and friends, multilingual staff, using gestures, or relying on “receptive multilingualism” [16–18]. While common and understandable, these approaches carry risks, including translation errors, omissions of critical information, and breaches of confidentiality [2, 18, 19]. The availability of qualified interpreters is often constrained by logistical, financial, and legal challenges, as well as shortages for languages of lesser diffusion [12, 13, 20, 21].

Technological tools, defined here as digital tools designed to facilitate direct, real-time communication between service users and providers, have emerged as a potential stopgap. These tools include online dictionaries, pre-translated fixed-phrase applications (apps), machine translation apps, both voice- and text-based systems, but explicitly exclude human-mediated digital solutions like video-interpreting. When accessible, such tools can offer flexible and immediate solutions, though their effectiveness varies with context, language, and complexity of information [22, 23]. Reported challenges include translation errors, limited phrase libraries, and the inability to capture emotional nuances, which may be crucial for mental healthcare interactions or similar consultations. Additionally, these tools must navigate complex ethical concerns, including data security [22, 24, 25].

Despite these challenges, technological tools may provide cost-effective, scalable solutions for basic communication in multilingual settings, reduce reliance on interpreters and ad-hoc solutions, and enable real-time communication in emergencies or resource-limited contexts [19, 26]. Their adoption and effectiveness depend on factors such as cultural and context adaptability, data security, and the availability of region-specific languages [22].

We have limited understanding of how technological tools can support communication in mental healthcare settings. Many existing studies have focused on general medical contexts or emergency care. Only a few studies have specifically explored the use of these tools in mental healthcare, and even fewer have employed qualitative research approaches to capture the nuanced, real-world experiences of diverse actors. By focusing on translation technologies, where the tools serves as the sole mediator in service user-provider communication, this study examines the experiences, attitudes, needs, and suggestions for the use of technological technologies to overcome language barriers in a mental healthcare setting, drawing on qualitative interviews with multiple actor groups across five diverse country contexts.

## Methods

### Research design

This study is part of a larger interdisciplinary research project, “Multilingualism in providing mental healthcare to migrants – needs, resources and practices” (MiM2M), and a qualitative study that identifies the barriers and resources in providing mental healthcare to migrants with a focus on multilingualism in psychosocial healthcare settings. The research employs a qualitative design, using semi-structured interviews to capture the diverse perspectives of healthcare providers, interpreters, service users, supporters, and stakeholders. Data were collected across five countries, reflecting diverse linguistic and healthcare contexts. The study followed the consolidated criteria for reporting qualitative research (COREQ) to ensure transparency and rigour in the reporting of methods [27]. An additional file shows this in more detail [see Additional file 1].

The study was conducted by a multinational and interdisciplinary team, including psychologists, linguists, medical researchers, and epidemiologists, including both early-career and senior researchers, with prior training and experience in qualitative interviewing and data analysis. The team consisted of both male and female researchers, bringing diverse professional backgrounds in migration, health, and languages, as well as varying degrees of fluency in the languages spoken by participants.

### Setting

The study was conducted in five countries: Germany, South Africa, Romania, the Netherlands, and China, reflecting a wide range of linguistic landscapes, healthcare infrastructures, and migration experiences.

### Participants and recruitment

Participants were recruited using a combination of purposive and snowball sampling methods, including direct email invitations, phone calls, distribution of flyers and posters, and engagement at community events, to capture the perspectives of multiple actors. All participants were required to be at least 18 years old. The inclusion criteria for each participant group were as follows:

- Healthcare Providers (HCP): Required to be professionally active in the (psychosocial) healthcare field, with training in medicine, psychology, social work, or a related healthcare sector. They needed to work regularly with service users experiencing mental health problems and to interact within language discordant situations at least once per week.
- Interpreters (IP): (Qualified) interpreters with experience in healthcare settings, as well as multilingual staff (e.g., nurses, security personnel) who have acted as informal interpreters. Potential interpreter participants needed to have completed at least 10 interpreting assignments, formal or informal, to be eligible.
- Service Users (SU): Participants were required to have at least one of the following:
  - Experience as users of psychosocial services, either currently or in the past, with self-reported experiences of language barriers in healthcare contexts.
  - Experience of attempting to access health or other services for mental healthcare but not having accessed these, owing to language and/or other barriers.
- Supporters (SUP): Participants were required to have experience supporting individuals with limited language proficiency in the dominant regional language in accessing psychosocial healthcare. This group included family members, friends, or acquaintances, among others.
- Stakeholders (SH): Participants were required to have expertise in the fields of migration and/or language barriers in healthcare, with roles potentially including policymakers, educators, community organisation representatives, government officials, or researchers.

### Data collection

The researchers developed semi-structured interview guides with specific questions tailored to include strategies for overcoming language barriers using technology. While the topical structure of the interview guides was similar, the wording of the questions was adapted to the specific participant group, ensuring both comparability and clarity within each context (Table 1). The interview guide also included a short sociodemographic survey to capture background information, such as age, gender, professional role, and years of experience. The interview guide was pilot-tested to ensure clarity and relevance.

**Table 1:** Technology-specific example questions from the service users’ interview guide

• What strategies do you use to overcome the language barrier?
• Was a solution offered by the health care institution to resolve the difficulty you were experiencing?
• Another strategy is to use technological tools, such as mobile phones, tablets, apps (for example), that can automatically translate languages.
• To get a clearer picture, I would like you to recall a situation where you have used a technological tool to overcome a language discordance. Could you describe this particular situation in detail?
• If you have not had any experience of using technological tools to overcome language barriers - can you give any reasons for this.
• Looking ahead, technological tools could be a way to overcome language barriers. In your opinion, what will be needed in order for this solution to be more integrated into health care services in the future?
• What knowledge or skills do providers/clients need in order to communicate with technological tools to overcome language barriers?

The interviews were conducted primarily in one-on-one settings (thus, dyadic interaction), with exceptions when an interpreter was present (triadic interactions), in the participants’ preferred language and lasted from 30 to 100 min. In South Africa, most interviews with service users and supporters were conducted with an interpreter to facilitate communication between the participant and interviewer. Data collection took place between November 2023 and December 2024, coordinated independently by the researchers of the five country teams. Interviews were audio-recorded, transcribed verbatim, and translated into English where necessary, to facilitate cross-country analysis. To ensure data quality, the involved researchers followed agreed-upon standardised procedures for transcription and data extraction.

### Data analysis

Data analysis was conducted by AK (female, M.Sc. Epidemiology, qualitative research expertise, fluent in German and English), in coordination with LP (female, B.Sc. Psychology, basic qualitative research experience, fluent in German and English), and close consultation with SHR, TT, and MM, employing Braun and Clarke’s thematic analysis approach [28]. This approach involved six phases: familiarisation with the data, generating initial codes, searching for themes, reviewing themes, defining and naming themes, and producing the final report.

Given varying data collection timelines across participating countries, we initially coded datasets by country. The coding process followed a hybrid approach, beginning deductively with broad categories based on the research objectives and interview guide categories (e.g., experiences, attitudes, needs, suggestions), while allowing for inductive codes and themes to emerge as the analysis progressed. A codebook was created and continuously updated as new insights emerged, ensuring flexibility and consistency across the diverse datasets.

To ensure inter-coder reliability, team members (AK and LP) cross-checked coding, and resolved discrepancies through consensus during regular analytic meetings, thereby promoting consistency across country teams and datasets. Once the initial coding of country-specific datasets was completed, the collection of data was gradually merged into a growing cross-country dataset, allowing the researchers to identify broader, cross-contextual themes that capture both shared and context-specific perspectives. To aid interpretation, we considered how contextual factors, such as individual experiences, institutional practices, and systemic conditions, might shape the use of translation technologies [29]. Data were analysed using ATLAS.ti (version 25) and MaxQDA (version 2020).

### Ethics approval and consent

All study procedures were performed according to the guidelines of the Declaration of Helsinki and ethical approval for this study was obtained from the respective ethics committees in all participating countries. In Germany, approval was granted by the Local Psychological Ethics Committee at the Centre for Psychosocial Medicine, University Medical Centre Hamburg-Eppendorf (06.11.2023, ID: LPEK-0672). In Romania, approval was issued by Babeş-Bolyai University (20.10.2023, ID: 15.118), and in the Netherlands by the Faculty Ethics Assessment Committee of the Faculty of Humanities at Utrecht University (26.02.2024, ID: FEtC-H 23-158-03). The South African component was approved by the Social and Behavioural Sciences Research Ethics Committee at Stellenbosch University (26.09.2023, ID: 28367), and the study conducted in China received approval from the Monash University Human Research Ethics Committee (11.12.2023, ID: 40906). All individual participants of this study provided informed consent prior to participation, and country teams adhered to ethical and data protection standards throughout the research process.

## Results

Most emergent themes occurred across participant groups and countries, and therefore the results are presented jointly while indicating differences and similarities when relevant. We begin by briefly outlining enabling conditions that shape whether translation technologies can be used at all, before examining institutional and individual experiences across the main- and sub-themes. The section begins with a description of the study sample, which is followed by the thematic analysis findings.

### Sample description

The total sample is 175 participants across five countries: Germany (*n* = 46), South Africa (*n* = 49), Romania (*n* = 34), the Netherlands (*n* = 23), and China (*n* = 23). The sample comprised a diverse range of actors (see Table 2).

A total of 100 participants identified themselves as female, 67 as male, while a small subset preferred not to disclose their gender. Ages ranged from 20 to 73 years, with a mean age of 40.2 years (SD = 12.7). At least 112 participants indicated that they had been born abroad, illustrating the linguistic and cultural diversity of the sample.

Participants reported a range of educational levels, with Master’s degrees (*n* = 26) and Bachelor’s degrees (*n* = 22) most frequently cited, alongside individuals holding PhDs, high school diplomas, or vocational qualifications. The most commonly reported professional roles were interpreter, psychotherapist, and various designations in social and mental health services.

**Table 2:** Study sample categorisation

	Germany	South Africa	Romania	Netherlands	China	Total
Healthcare personnel (HCP)	8	4	7	4	4	**27**
Service Users (SU)	10	20	11	4	9	**54**
Supporters (SUP)	12	14	5	6	1	**38**
Interpreters (IP)	8	7	6	5	6	**32**
Stakeholders (SH)	8	4	5	4	3	**24**
**Total**	**46**	**49**	**34**	**23**	**23**	**175**

Across countries, participants noted that certain enabling conditions need to be in place before translation technologies can be meaningfully used, for example, reliable electricity, internet or mobile data, and access to suitable devices. In South Africa, several accounts described intermittent power and concerns about device security. Connectivity constraints were also reported in rural or underserved areas in other countries. By contrast, in contexts with broadly reliable infrastructure, participants could focus more on questions of accuracy, suitability, and workflow integration. This brief macro-context helps to explain why the depth of usability reflections may have varied across countries and participant groups, and why some participants described only improvised or sporadic use.

The thematic analysis of participant reports across the five countries revealed six overarching themes, each comprising several sub-themes (Table 3). These themes reflect shared experiences and perspectives regarding the use of technological tools to overcome language barriers in mental healthcare. Given the scope and depth of the dataset, spanning multiple countries and participant groups, this section presents the overarching patterns that emerged consistently across these contexts. Despite many similar themes, subtle differences in emphasis were noted in all countries and across participant groups. For instance, concerns about data protection were particularly salient among participants of the German sub-sample and among healthcare providers across the five countries. Where relevant this report indicates such contextual or group-specific nuances. However, the emphasis remains on synthesising cross-cutting insights that speak to the broader challenges and opportunities of using technological tools in multilingual mental healthcare settings. An additional file presents the binary presence of themes and sub-themes in a cross-country and cross-participant comparison [see additional file 2].

**Table 3:** Identified main- and sub-themes

Main theme	Sub-themes
Perceived effectiveness and limitations of technological tools	- Limited effectiveness - Context-specific suitability
Cultural, linguistic, and social fit	- Cultural and contextual suitability - Digital literacy and generational divide
Human aspects of communication and care	- Empathy and emotional connection - Trust and relational dynamics
Ethical and attitudinal dimensions	- User-based reflections and perspectives - Ethical considerations
Implementation and improvement pathways	- Technical improvements - Integration into existing care and workflows - Policy, training, and institutional support
Access barriers	- Infrastructure - Practical constraints to tool use

### Perceived effectiveness and limitations of technological tools

#### Limited effectiveness

Participants across countries and participant groups noted that while technological tools can support multilingual communication, their overall effectiveness is limited, particularly due to issues of translation accuracy and loss of nuance. These concerns were especially prominent in relation to specialised or sensitive terminology, such as medical or mental health vocabulary.

An interpreter from the Netherlands emphasised the potential inaccuracy of machine translation: *“There are mistakes in the translation*,* and you have to go over it again and again to convey the idea 100%. The translator doesn’t help at all.” (NL_IP_06)*.

Several participants described how even minor translation errors can carry significant risks in healthcare communication and therapeutic decisions. A supporter noted: “*Patients may agree to procedures without fully understanding the risks or alternatives. […] These small details can get lost in translation. This can be dangerous*,* especially in urgent situations where treatment is delayed or decisions about care are misunderstood.” (CH_Sup_01)*.

Others highlighted the difficulty of conveying precise meaning through translation tools. For instance, a service user recounted: *“I typed in phrases like ‘I have pain in my stomach*,*’ ‘It hurts when I eat*,*’ and ‘What should I do next?’ […] There were moments when the translations were not perfect*,* leading to slight misunderstandings. For instance*,* when discussing dietary restrictions related to my condition*,* some nuances were lost in translation.” (CH_SU_04)*.

Additionally, tools relying on pre-translated phrases or literal machine translation were often considered inflexible or insufficient for accurate communication. The inability to adapt language style, tone, or indirect phrasing often contributed to confusion and misinterpretation, especially when the original input was more complex than simple yes/no or symptom phrases.

Participants described pragmatic use of tools, but expressed a consistent need for human cross-checking or clarifying translations, either through repeated questioning or switching to another language, which undermined the efficiency and reliability of these tools in dyadic interactions.

#### Context-specific suitability

Participants consistently emphasised that the suitability of technological tools depends heavily on the specific healthcare context in which they are used. Rather than being seen as universally effective, tools were described as conditionally helpful, with usefulness varying across types of interaction.

Many reported that digital tools could assist with simple, structured exchanges, such as collecting administrative information, confirming identity, or asking routine intake questions. As one interpreter explained: *“[…] for initial conversations*,* […] Year of birth*,* place of birth. For things like that*,* […] And then it is helpful when we are unable to communicate at all.” (GER_IP_07)*.

Participants across countries agreed that tools were far less effective in situations requiring complex medical conversations, especially those involving diagnosis, treatment planning, or risk communication. A stakeholder reflected: *“While translation technology has improved a lot*,* it is not always 100% accurate*,* particularly with complex medical words or difficult conversations.” (CH_SH_3)* In emergencies, where time is limited and no interpreter is available, several participants described using tools as a “last resort” to establish basic communication. This perspective was particularly shared by healthcare providers working in urgent or acute care settings, who often face language barriers they could not anticipate under time pressure. A healthcare provider noted: *“It usually happens in emergency situations*,* especially at night or when there is no one available to help with translation. In those cases*,* we rely on tools like Google Translate to communicate with patients.” (RO_HCP_06)*.

Some providers described adapting their own communication style to make the tools more effective, for instance, by simplifying vocabulary or avoiding idiomatic expressions. One participant explained: “*They’re not perfect*,* but they can help convey basic information or understand simple responses. […] I use simple terms*,* speak slowly*,* and avoid idioms or complex phrases.” (CH_HCP_02)*.

These accounts reflect a pragmatic, often reluctant, and improvised use of translation technologies, not as a first-line solution, but as a stopgap measure when other resources are unavailable. Their perceived usefulness hinged not only on the tool itself, but also on the clinical context, the urgency of the situation, and the ability to simplify communication.

### Cultural, linguistic, and social fit

#### Cultural-contextual suitability

Participants from various countries and groups emphasised that effective translation requires more than literal accuracy. Digital tools must also account for cultural norms, idiomatic expressions, medical metaphors, and linguistic diversity. Even technically correct translations often fail to capture indirect meanings or sociocultural nuances, leading to awkward or misleading communication.

A healthcare provider noted: *“They do one-to-one translation and not this cultural background*,* nothing is translated at all. And that sometimes results in a strange translation.” (GER_HCP_01)*.

Idioms and indirect speech were particularly challenging for machine translation tools. An interpreter stressed the need for cultural awareness to complement language translation: *“The most important aspect for these tools is incorporation of cultural awareness*,* not just language translation. It will bridge cultural gaps in addition to linguistic ones.” (CH_IP_5)*.

Participants also highlighted gaps in language coverage, especially for dialects and languages of lesser diffusion. A healthcare provider shared that the translation app their employer provided didn’t represent the diverse language needs of their patients: *“Most of the refugees are*,* as I said*,* from Afghanistan*,* Syria or I mean Ukraine*,* of course*,* many of these languages don’t appear at all.” (GER_HCP_03)*.

Others described inaccuracies stemming from diacritical marks used in writing. As a supporter recounted: *“One of the questions was: ‘What is your religion?’ But it was misread as ‘What is your debt?’ […] It was all because of one small diacritical mark that completely changed the meaning of the word.” (NL_SUP_03)*.

Concerns were also raised about bias in artificial intelligence-based tools, which may reflect or reinforce dominant cultural assumptions. As one stakeholder put it: *“Those tools*,* because they copy what they find out there on the Internet*,* they copying cultural bias. And they are not tailored to the specific linguistic nuances of that particular context.” (ZA_SH_02)*.

These findings highlight a core limitation: without cultural and linguistic adaptability, translation tools risk misrepresentation, confusion, or exclusion, particularly in sensitive mental healthcare contexts.

#### Digital literacy and generational divide

Participants emphasised that digital literacy significantly influences who can effectively use translation tools, frequently noting generational differences. Older adults, both service providers and service users, were often described as less confident or more reluctant to engage with digital solutions. As a healthcare provider shared: *“Older doctors may be less open to adopting new technologies. They often distrust these tools and prefer traditional methods*,* even if those methods are less effective.” (RO_HCP_01)*.

However, participants also noted that digital fluency does not always align with education level or age. A supporter reflected: *“These people know how to handle a mobile phone. They might not know how to write with pen and paper*,* but they can write on WhatsApp.” (NL_SUP_05)*.

This diversity in digital competence suggests that training and tool usability must be adaptable to accommodate a wide range of users. Tools should be intuitive, with minimal learning curves, and developers should consider accessibility features such as voice input or visual icons to support users with limited literacy or formal education.

Participants’ reflections highlight the importance of context-sensitive onboarding and optional institutional guidance, particularly for older users or those unfamiliar with mobile apps. Designing solutions that cater to diverse user needs, rather than just tech-savvy users, will be crucial for the equitable implementation of these solutions.

### Human aspects of communication and care

#### Empathy and emotional connection

Participants across countries emphasised the limitations of translation technologies on an emotional level. While tools may enable basic understanding, they were widely seen as unable to convey empathy, warmth, or emotional nuance. This sub-theme reflects participants’ emphasis on the affective quality of communication and the comfort that comes from being emotionally understood. Human interpreters were often described as sources of emotional safety, able to recognise distress, adjust tone, and offer reassurance. As one interpreter noted: *“Just the presence of a person*,* a living person who feels and thinks for themselves*,* it creates a different kind of trust.” (GER_IP_02)*.

Several participants described how devices felt impersonal or emotionally inadequate, particularly in sensitive or vulnerable moments. A supporter explained: *“Maybe with more intimate things*,* […]. Like my mother*,* for example*,* when she needs me. I think she just feels more comfortable that I convey that. And talking into this device*,* I don’t know.” (GER_SUP_AR_04)*.

Still, some valued the privacy or reduced emotional exposure that technology can offer. One service user shared: “*Actually*,* I find online translator apps better than having someone physically present.” (GER_SU_AR_06)*.

These mixed views illustrate that many participants perceive emotional nuance as a distinctly human strength, one that current technologies are not equipped to replicate. While tools can support information exchange, many felt they fall short of providing the empathetic presence essential for therapeutic care.

#### Trust and relational dynamics

Participants frequently mentioned that the use of technological tools can disrupt the natural flow of interaction and impact the interpersonal dynamics essential for building trust in mental healthcare. Unlike solely human communication, which they perceived as enabling spontaneity, rhythm, and mutual adjustment, device-mediated communication was often experienced as rigid or fragmented. A healthcare provider noted, *“Then certain things get lost. Like relationships. Because you follow a strict sequence*,* but relationships*,* humour*,* warmth*,* etc. fall by the wayside. And I think that is what work in psychosocial communication thrives on*,* […]*,* 80% is relationship.” (GER_HCP_05)*.

Others found the constant handling of a device awkward or intrusive: *“I find it annoying because you always have a mobile device in your hand and you’re passing it back and forth.” (GER_HCP_04)*.

Despite these concerns, some participants described adaptations and hybrid strategies that helped preserve interactional flow. A service user appreciated not needing a third person in the room, and several participants supported combining human interpreters with technology for clarification or support. Some saw having access to an app as empowering, allowing service users to engage more independently and retain control over their communication. As one interpreter suggested, *“Let’s say you called an interpreter to the emergency room who didn’t have time to prepare. You allow them to use technology. They can check definitions in the other language to make sure it doesn’t pass on erroneous information […].” (RO_IP_01)*.

These perspectives highlight the nuanced role of technology in relational dynamics. Trust is not just about understanding content, but also about how communication unfolds, the ability to build rapport, coordinate turns, and adjust in real time. While tools may offer practical support, they can also introduce friction into relational processes that are vital to effective care.

### Ethical and attitudinal dimensions

#### User-based reflections and perspectives

Participants shared ambivalent but evolving attitudes toward translation technologies. Many expressed initial scepticism but described gradually adopting a pragmatic stance, using tools as needed, particularly in fast-paced or low-resource settings. These reflections were less about ideal use and more about adapting under imperfect conditions. A healthcare provider shared: “*But it’s also better than nothing*,* of course*,* there are mistakes*,* but again*,* there’s the context*,* I know what he means or what he wants.” (GER_HCP_02)*.

Several participants emphasised the conditional use of translation apps, depending on the urgency, topic, or availability of alternatives. Rather than expecting flawless performance, many participants emphasised “good enough” communication when no better option was available. Especially in frontline or emergency care, providers valued the independence and immediacy of translation apps, despite their known limitations.

Some participants also described using tools as a backup or recognised them as a helpful supplement when interpreters were present. An interpreter shared: *“I have sometimes used the app myself*,* for example when I didn’t know about an illness. So I looked it up to be really sure.” (GER_IP_03)*.

These reflections highlight participants’ situational and critical engagement with translation tools. Rather than fully endorsing or rejecting them, most described a form of conditional acceptance, viewing the tools as helpful in specific scenarios, but not reliable in all situations. Their attitudes were shaped less by general beliefs and more by practical experience, with many using workarounds, simplifying language, or combining tools with human input to achieve their goals.

#### Ethical considerations

Participants from all countries and groups raised ethical concerns related to the use of translation technologies in mental healthcare, particularly regarding data protection, informed consent, and institutional responsibility. These concerns were especially prominent among the German sample and among healthcare providers across countries.

The most frequently cited issue was data security, specifically, uncertainty about how and where sensitive information is stored or processed. In particular, participants from Germany, raised concerns related to the GDPR and the risks associated with using commercial apps, such as Google Translate, without proper safeguards. One service provider noted: *“Sharing sensitive data such as medical and health issues with Google is something I am skeptical about. […] it is a good idea to be suspicious about what happens next with the data processing.” (GER_HCP_06)*.

Informed consent was another topic. Participants emphasised that service users should be made aware of how their data is handled and the potential risks associated with using translation apps, especially when disclosing traumatic or sensitive experiences. As one stakeholder put it: *“They are telling the most horrible stories that rape stories or and you’re not really sure what’s gonna happen with the data or even if it’s secure. Does the patient feel secure knowing that there can always be a data leak?” (NL_SH_05)*.

Beyond privacy issues, some participants warned against over-reliance on technology, raising concerns that institutional use of apps without guidance or oversight could erode professional responsibility, bypass critical reflection, and be detrimental to existing structured use of professional interpreters. Several questioned whether current systems were doing enough to regulate or support the ethical use of these tools in mental healthcare and healthcare contexts overall.

Overall, these reflections demonstrate that ethical concerns, particularly regarding privacy, consent, and technological dependency, can serve as barriers to adoption. They also underscore the need for secure, transparent, and contextually appropriate tools that align with both institutional standards and users’ rights.

### Implementation and improvement pathways

#### Technical Improvements

Participants from various countries and groups provided numerous concrete suggestions for enhancing translation technologies in mental healthcare.

Participants emphasised improvements in effectiveness and accuracy, noting the need for tools to handle complex and context-specific language more reliably, particularly in mental health and medical settings. Suggestions included integrating specialised dictionaries, standardising terminology, and offering clearer outputs. Some participants recommended features that would allow users to verify or cross-check translations, especially in high-stakes conversations. As an interpreter noted: *“The translation tool must be able to handle complex medical terms with great accuracy. Maybe we need to develop a special medical dictionary.” (CH_IP_06)* A healthcare provider reflected: *“It’ll still be important for humans to oversee and intervene […]. Future solutions should make it easy for healthcare professionals to verify and refine machine translations.” (CH_HCP_02)*.

Participants also called for greater efficiency, particularly through real-time, voice-based translation for use in urgent care and fast-paced settings. They envisioned tools that require minimal manual handling and input, such as one-tap voice translation or hands-free functionality: *“There should be a single button you press to start speaking*,* and the app should translate in real-time.” (RO_HCP_06)*.

Enhancing learnability and ease of use was seen as another priority. Tools should be intuitive and easy to use for both providers and service users, regardless of digital literacy. Clear interfaces, simplified navigation, and low onboarding requirements were seen as key design priorities. An interpreter described: *“The tool should have a straightforward and intuitive interface that allows healthcare providers and patients to navigate easily without extensive training.” (CH_IP_04)*.

To accommodate diverse needs, participants suggested improving accessibility and multimodality by incorporating multiple input and output options, such as voice, text, and visual elements. Several participants also highlighted the importance of offline functionality and compatibility with existing hardware. A service user envisioned: *“The person speaks and the app translates. If it is really precise and correct*,* it will be perfect.” (GER_SU_AR_01)*.

Another key priority was cultural and linguistic adaptability, including **i**mproved support for languages of lesser diffusion, dialects, and culturally nuanced communication. Participants called for tools that go beyond literal translation and reflect regional variation and cultural particularities. An interpreter shared: *“The tool should also incorporate cultural nuances and sensitivities*,* ensuring that translations are not only linguistically accurate but also culturally appropriate. The tool should support a lot of languages*,* including dialects and regional variations*,* to accommodate diverse patient populations.” (CH_IP_04)*.

While addressed in more detail in sub-theme 4.2 on ethical considerations, participants reiterated that privacy protections should be built into the tools themselves. This would include transparent data policies, secure processing, and alternatives to commercial platforms. A healthcare provider said: *“More data protection compliant solutions are fundamentally necessary.” (GER_HCP_06)*.

Together, these suggestions reflect a desire for tools that are not only technically robust but also practical, user-centered, culturally responsive, and ethically sound.

#### Integration into existing care and workflows

The successful use of translation technologies depends not only on their technical features but also on their seamless integration into healthcare workflows and systems. When tools are poorly integrated or absent from institutional infrastructure, providers often mention relying on personal smartphones and improvised solutions, raising concerns about consistency, security and efficiency.

Several participants described a mismatch between available hardware and communication needs. For example, while many healthcare facilities had tablets or digital documentation systems, these often lacked built-in or authorised translation software. A healthcare provider shared: *“We have a tablet for recording*,* billing*,* and creating reports*,* but it doesn’t have a communication app or anything like that” (GER_HCP_06)*.

Participants envisioned more streamlined, embedded solutions, such as translation tools pre-installed on clinical devices, accessible across care settings and departments. Easy access across locations, not just in designated areas, was viewed as essential to respond flexibly to communication needs as they arise: *“It would be great if they could be available on different devices and be easily accessible throughout healthcare facilities.” (CH_SU_NI_01)*.

Hands-free, wearable technology and voice-activated features were considered particularly useful for maintaining clinical flow, especially in urgent care contexts where mobility and speed are critical. One provider explained: *“And if*,* for example*,* the microphones are good enough*,* you can just hold it in your hand and then you can speak into it [the documentation tablet]” (GER_HCP_04)*.

Participants also emphasised the importance of aligning translation tools with existing clinical routines and documentation systems to minimise workflow disruptions and cognitive load. Integration was viewed as more than a technical matter; it required organisational commitment to ensure that tools are functional, accessible, and usable as part of routine care.

#### Policy, training, and institutional support

Across countries and participant groups, there was broad agreement that to use translation technologies effectively in healthcare requires support structures, including clear policies, institutional guidance, safeguards, and appropriate training. Without formal guidance, decisions about which tools to use, when to use them, and how to manage associated risks are frequently left to individuals. Many described relying on personal devices or unregulated apps in the absence of approved alternatives or clear protocols. As a healthcare provider explained: *“It’s mostly up to the staff to figure out what to use*,* but there’s no official guidance or support.” (RO_HCP_06)*.

Several participants called for policy-level interventions, including establishing basic standards for tool use, data protection, and clinical appropriateness. Some suggested that translation support should be integrated into broader language access strategies, backed by public funding or regulation, to ensure effective implementation.

Training needs were described as practical and targeted, rather than extensive. Participants asked for short introductions that highlight common risks, usability limits, and appropriate use cases. In a healthcare provider’s words: *“You don’t have to do two days of training […] you just have to find out more about where the stumbling blocks are […]” (GER_HCP_06)*.

Several participants, predominantly healthcare providers and stakeholders, emphasised that the primary responsibility for providing and learning how to use translation tools should lie with healthcare institutions and professionals. As one provider put it: *“For patients*,* I think it would be unreasonable to expect them to adapt to using such tools. It’s our responsibility to provide healthcare without imposing conditions on them. The burden falls on us as providers. […]” (RO_HCP_01)*.

At the same time, participants emphasised the importance of informing service users about the limitations and risks associated with these tools. Education and awareness-raising were seen as essential for enabling service users to make informed decisions about whether and how to engage with translation technologies. An interpreter explained: *“Well*,* I think it’s not just professionals*,* patients or clients should also be informed about these risks*,* […]Yes*,* it’s not perfect*,* it’s not 100% what’s being used here.” (GER_IP_02)*.

In summary, participants called for clearer policies, practical training, and shared understanding among all users. Without these kinds of support, the use of translation tools remains inconsistent and risks reinforcing uncertainty, rather than improving access.

### Access barriers

#### Infrastructure

Participants in all country contexts described infrastructure-related barriers that limited the reliable use of translation technologies. These included unstable electricity and poor internet connectivity, both of which directly affected whether tools could be used at all.

Participants from the South African sub-sample noted that regular power outages and unstable power supplies can hinder consistent access to, and thus, their reliance on digital tools. As a service user shared: *“[…] sometimes it is limited due to problem of electricity or network.” (ZA_SU_15)*.

Internet access was also a commonly cited issue. In some settings, especially in rural areas, poor connectivity rendered digital tools unreliable or entirely unusable, as an interpreter recounted: *“Issues such as poor internet connectivity can hinder the effectiveness of these tools.” (CH_IP_04)*.

These reflections highlight how infrastructure shapes the feasibility of using translation technologies in everyday care. Even when tools are available, they often remain inaccessible in practice due to poor electricity supply and connectivity. This reality disproportionately affects under-resourced or rural settings.

#### Practical constraints to tool use

Participants highlighted that, similar to infrastructure constraints, practical issues like monetary cost of devices, apps, and internet access can be a major barrier to translation technology use, and more so for users in under-resourced settings. Some tools require paid subscriptions or data usage, making them inaccessible to those with limited financial means. An interpreter emphasised: *“Yes*,* preferably free of charge*,* so accessible to everyone.” (GER_IP_01)*.

Concerns about the physical security of devices in the healthcare environment were raised in the South African sub-sample. They mentioned possibilities of damage, loss or theft of tablets or smartphones. A healthcare provider shared: *“Sad to talk about it*,* but some people might*,* even workers*,* might remove it from the client*,* you know*,* and steal it.” (ZA_HCP_02)*.

Healthcare providers noted the lack of institutional approval or even restrictions on specific technologies as a potential barrier. For example, some workplaces prohibit the use of translation apps due to privacy concerns. A healthcare provider recounted: *“Because I think in our company it’s not allowed because of the privacy.” (NL_HCP_05)*.

Finally, time pressure in healthcare settings was mentioned as a key barrier to using translation technologies. Navigating translation apps, clarifying misunderstandings, or troubleshooting technical issues often required more time than was available in clinical encounters. A service user shared their experience: *“They don’t have time. They don’t have time for you for that” (ZA_SU_31*) An interpreter recalled that some service providers refuse to treat service users with the help of a translation app: *“Because of the extra explanations generated by communication problems […] some have said no more apps*,* no more patients without an interpreter.” (RO_IP_08*).

Taken together, these reflections point to a range of practical constraints that limit the feasibility of using translation tools in daily care.

## Discussion

### Contextualising the study

This qualitative study offers a cross-country perspective on the experiences and perceptions of translation technologies in multilingual mental healthcare among service users, interpreters, healthcare providers, supporters, and stakeholders. While translation technologies are increasingly viewed as promising tools to address language barriers in healthcare, our findings highlight that context, infrastructure, relational dynamics, and institutional support influence their effectiveness and adoption.

Although most themes and sub-themes appeared across all countries and participant groups, certain issues were emphasised with greater intensity in specific national contexts. For instance, data protection and privacy concerns were particularly prominent among the German and healthcare provider sub-samples. Infrastructure challenges, such as unstable electricity and internet dependency, were more commonly raised in the South African sub-sample. These variations should not be interpreted as binary differences, but rather as differences in emphasis and salience, shaped by local policy environments, healthcare infrastructures, and socio-political contexts. As outlined in the brief macro-context at the start of the Results, differences in enabling conditions such as electricity, connectivity, and device availability help explain variation in the depth and nature of participants’ reflections across countries and groups. Recognising these contextual nuances is essential for designing translation solutions that are not only technically effective but also responsive to diverse regional needs.

### Usability in practice

This study’s participants described translation tools as applicable in structured or routine interactions, such as collecting demographic data or scheduling appointments, but often unreliable in more complex, high-stakes situations.

Concerns about translation accuracy were widespread, particularly in emotionally sensitive or clinically complex contexts, such as mental health consultations. Delfani et al. [30] highlighted the challenges that machine translation systems face in mental health contexts, where language is often ambiguous and deeply context-dependent, noting that errors can compromise safety and quality of care [30]. Similarly, Brewster et al. (2024) reported on inaccuracies in machine-generated discharge instructions contributing to confusion about medication and follow-up, reinforcing the need for such tools to complement, rather than replace, professional interpreters in critical or culturally sensitive interactions [31]. Our data and the literature confirms, that the risk of miscommunication is not hypothetical but a tangible barrier to trust and reliance on these tools in complex care encounters.

These concerns reflect a broader pattern in the data, indicating that the perceived suitability of translation tools is highly context-specific. Tools were often used pragmatically but cautiously, valued for enabling some degree of communication, yet rarely considered reliable for conveying complex or nuanced meaning. This aligns with implementation science frameworks, which emphasise the importance of context and adaptability in technology adoption [32, 33]. The “conditional acceptability” of translation tools in emergencies reflects principles of adaptive implementation, where users modify tools to fit their immediate needs, often in the absence of formal guidelines. For example, Damschroder et al.’s (2009) Consolidated Framework for Implementation Research highlights the role of the “outer setting” (e.g., urgent care demands) and “intervention characteristics” (e.g., tools perceived adaptability) in shaping adoption patterns [32].

Readiness to use translation technologies also varied across different clinical settings. A notable trend in the data was the higher openness among service providers primarily working in urgent or emergency care contexts, who described tools as a “last resort” when no interpreter was available. While this pragmatic use reflects broader digital health patterns, where urgency drives adaptive tool use despite limitations, it introduces a critical tension in clinically or relationally sensitive situations (e.g., trauma disclosure, risk assessment) [33]. The immediacy of care needs must be weighed against the potential for miscommunication, misdiagnosis, or compromised trust, especially when tools lack the nuance or cultural adaptability required for high-stakes interactions. Such patterns are echoed in several studies that highlight the widespread use of translation technology in emergency and prehospital care, where structured language support is often unavailable and providers rely on fast, stopgap communication methods [25, 26, 34]. These findings reflect how individual, institutional, and systemic factors, such as provider digital literacy, workflow integration, and resource constraints, interact to shape the usability of translation tools in mental healthcare.

### Cultural and linguistic fit

Participants consistently noted that translation tools often fail to capture cultural nuances, idiomatic language, or indirect expressions. Even technically accurate translations were frequently perceived as awkward, misleading, or missing their intended meaning. This disconnect was particularly problematic in mental healthcare, where communication often involves layered, emotionally charged, or context-dependent language. These findings reinforce prior research on the limitations of machine translation in handling culturally embedded communication [30, 35, 36]. Participants further raised concerns about the limited range of languages covered in existing tools, particularly for languages of lesser diffusion. These challenges echo broader findings in the literature regarding the structural limitations of translation technologies. Brewster et al. (2024) found that machine translation platforms, including Google Translate and ChatGPT, performed inconsistently across languages, with significantly lower quality in low-resourced languages such as Haitian Creole, compared to more widely used languages like Spanish and Portuguese [31]. Similarly, Delfani et al. [30] reported that machine translation tools struggled to accurately translate mental healthcare information into Arabic and Romanian, due to limitations in domain-specific terminology and syntactic complexity [30]. These findings underscore the pressing need for more multilingual and domain-specific training data to mitigate quality disparities and enhance inclusivity.

Participants in our study also emphasised the importance of culturally adaptive tools, not merely translating words, but also capturing culturally appropriate meanings and communicative styles. Without this, even well-intentioned use of technology can lead to alienation or misrepresentation. In line with previous research, our findings support the calls for participatory and co-design approaches that include linguistically diverse communities in the development process [19, 35, 37].

Taken together, these insights suggest that without structural investment in linguistic diversity, translation tools risk reinforcing existing inequities, performing well in dominant languages while underserving those who already face raised barriers to care. For these technologies to support equitable mental healthcare, linguistic and cultural adaptability must be treated as core components of usability, not peripheral enhancements.

### Relational and emotional dimensions

Participants widely agreed that human presence remains essential in mental healthcare communication, not only for understanding content but also for building trust, conveying empathy, and creating emotional safety. Many described translation tools as impersonal or emotionally inadequate, particularly in moments of distress, trauma disclosure, or therapeutic engagement. Even when tools enable basic understanding, they were seen as lacking the warmth, tone, and responsiveness that human interpreters can offer. This aligns with Bordin’s (1979) model of therapeutic alliance, which identifies the emotional bond between service user and provider as a cornerstone of effective therapy [38].

Research on interpreter-mediated psychotherapy further underscores this point, demonstrating that trust and empathy, critical components of the alliance, are fostered not only through non-verbal cues (e.g., eye contact, facial expressions) but also through cultural attunement and real-time emotional validation [39]. While translation technologies may allow for some non-verbal cues, they disrupt the temporal alignment between spoken words and emotional expressions. For example, delays in translation can create a lag between verbal content and non-verbal reactions (e.g., a smile or nod), making it harder for service users and providers to feel emotionally attuned. These concerns have also been raised in previous research, where participants expressed that translation technologies may diminish empathy in service user-provider interactions by failing to capture emotional nuance and relational subtlety [19, 40].

Participants also described how translation tools disrupted the natural flow of conversation. Handling devices, such as passing a phone back and forth or waiting for translations, often interrupts interactions and makes it harder to maintain a connection or focus on the other person. Such disruptions fragment the collaborative rhythm central to the alliance’s task and goal components [38].

Empirical research on the impact of translation tools on the therapeutic relationship between service users and providers remains very limited. While some studies mention empathy or interpersonal distance, we found no focused investigations into how these tools influence trust-building, rapport, or long-term provider-user relationships [19, 40].

### Ethical considerations

Participants raised concerns about the ethical implications of using translation tools in mental healthcare, particularly regarding data protection, informed consent, and institutional responsibility. Many expressed discomfort with commercial platforms like Google Translate, citing uncertainty about how user data is processed. These concerns highlight the need for clear institutional policies and healthcare-specific tools that ensure privacy, transparency, and informed use. Without such safeguards, translation technologies may compromise user confidentiality and place ethical burdens on individual providers. These findings align with existing research calling for secure, regulated platforms and stronger ethical oversight in the use of digital translation tools [22, 41, 42]. To uphold ethical standards, these technologies should complement, not replace, formal language support systems and be embedded within a framework of institutional accountability.

### Implications for practice

The successful implementation of translation technologies in mental healthcare depends not only on technical design but also on institutional support and practice-oriented approaches grounded in implementation science. The need for clear guidelines and protocols that specify when, how, and which translation tools should be used was emphasised. In the absence of such structures, healthcare providers and service users often relied on personal smartphones or improvised solutions, resulting in inconsistencies and increased risks related to accuracy, data protection, and the quality of care. These findings are echoed by Taylor et al. (2024), who emphasise that embedding clear guidance into clinical workflows is essential for aligning the use of translation tools with the broader goals of service user safety and effective communication [35].

Applying insights from implementation science, successful integration of translation tools requires attention to the “inner setting” (e.g., organisational culture, workflow integration) and “process” (e.g., user engagement, iterative feedback) to ensure tools are used effectively and equitably [32]. Greenhalgh et al. (2004) further highlight that innovations in healthcare are often adapted informally by end-users to fit their workflows, a pattern observed in our study where providers modified their use of tools in response to contextual constraints. Without structured support, such as institutional guidelines or training programs, these adaptions risk being inconsistent or inappropriate, potentially undermining communication in mental healthcare [33]. Rather than leaving decisions to individual discretion, organisational protocols should provide structure, accountability, and support for responsible implementation. Other scholars have similarly argued that guidelines must be context-specific, clarifying where and when translation tools are most appropriate. That implementation should be based on robust evidence of their effectiveness and limitations [22, 31, 35].

Learnability and accessibility were key considerations for participants in their reflections. They called for simple, clearly structured interfaces with minimal onboarding, as well as features like voice options or visual prompts to support users with lower literacy or digital literacy. These concerns align with research emphasising inclusive design and the need to accommodate diverse user competencies [19, 25]. Some studies have shown that visual or interactive features, such as icons, illustrated symptom scales, or guided prompts, can help explain abstract concepts, reduce the burden of repeated explanations, and support emotional connection in interactions [25, 43]. Additionally, features such as conversation history and saved translations were valued for supporting information retention and follow-up care [34, 44]. Participants stressed that tools must be usable by both healthcare providers and service users, across a range of digital competencies.

Beyond usability, participants highlighted the importance of integrating translation tools into existing clinical workflows and infrastructure. Rather than treating these tools as optional add-ons, institutions should embed them into commonly used systems, such as documentation platforms or wearable devices. Offline functionality and multimodal access (e.g., text, audio, visual) were considered essential to ensure equitable use across settings with limited internet access or unreliable power, as well as to enhance efficiency and effectiveness. These findings align with earlier research that advocates for context-aware deployment and technology that functions reliably in real-world conditions [35, 36].

The participants primarily assigned responsibility for effective translation technology implementation on healthcare systems and professionals. However, they also emphasised the importance of informing service users about how these tools work, including their capabilities and limitations. This dual approach, combining professional accountability with service user empowerment, was seen as key to promoting person-centred and ethically sound communication [26, 31, 41].

Access barriers, such as poor internet connectivity and power outages, limit the use of translation technologies. Participants described how unstable infrastructure rendered digital tools unreliable or unusable. These challenges align with previous research and underscore the need for offline-capable, context-adaptive solutions that do not rely on constant connectivity [22, 35, 36]. Additionally, financial constraints, including subscription fees, mobile data costs, and the absence of publicly funded or institutionally approved tools, hindered adoption for both service users and providers. Low-cost or open-access tools may help reduce these barriers by offering more equitable access across diverse environments [22, 35, 42].

Addressing these structural and practical barriers will require targeted investment in infrastructure, the development of publicly supported or open-access tools, and institutional policies that enable responsible and context-sensitive implementation. These tools should not be seen as a replacement for qualified interpreters, particularly in complex interactions. Without proper safeguards, there is a risk that such tools will be over-relied upon in settings where human interpretation is needed most, potentially compromising care quality and safety. Their integration into mental healthcare should therefore be approached with caution, guided by realistic expectations and grounded in a commitment to equity, clinical appropriateness, and person-centred communication.

### Implications for research

Our findings highlight several important knowledge gaps that should be addressed in future research. While much of the existing evidence has focused on emergency and general healthcare contexts, the specific demands of mental health remain underexplored. Prior studies and our findings show that many translation tools struggle to convey mental health terminology and emotional subtleties, particularly in less-resourced languages such as Arabic, Persian or Romanian [30, 31]. These challenges are closely tied to broader issues of linguistic equity. Many apps underperform in underrepresented languages due to the limited availability of multilingual training data and insufficient contextual adaptation. Without addressing these gaps, translation tools risk reinforcing existing disparities in access and quality of care. Future research should systematically evaluate the usability of translation tools across a broader range of languages and contexts, and support the development of domain-specific solutions that are both linguistically and culturally inclusive, as well as clinically appropriate.

Further, current evaluation frameworks tend to emphasise technical performance or general usability, often overlooking emotional, cultural, and ethical dimensions of tool use. At the same time, methods for assessing usability or similar outcomes remain highly heterogeneous across studies, limiting comparability and the development of evidence-based recommendations [31, 41]. Developing shared yet adaptable evaluation criteria would enable more robust comparisons across tools and settings, while ensuring that the specificities of mental healthcare are not overlooked.

Our findings also highlight the importance of participatory and co-design approaches. Including diverse actors has been shown to improve usability. Iterative feedback processes can help tools remain adaptable to evolving clinical needs, terminologies, and user preferences. At the same time, co-design must address ethical concerns, such as data privacy and translation bias [34, 37]. Interdisciplinary research involving mental health service providers, linguists, and developers is needed to ensure that the research and development of translation tools are both practically meaningful and socially responsible.

A critical area for future inquiry concerns the impact of translation technologies on therapeutic relationships. Despite frequent concerns about reduced empathy and disrupted interpersonal flow, there is a lack of research systematically examining how translation tools affect the therapeutic alliance. Studies are needed to understand when and how these tools may support, rather than hinder, trust and connection in healthcare relationships.

Practical implications of using translation tools, such as disruptions in communicative flow, additional cognitive load, and device handling during, warrant further empirical attention. As our participants indicated, such challenges can affect the fluidity of interaction and potentially detract from the delivery of person-centred care. Studies that focus on these real-world dynamics, including workflow disruptions and relational impacts, would offer valuable insight into the potential limitations and strengths of digital translation.

Together, these priorities underscore the need for a multifaceted and ethically grounded research agenda. Moving forward, research should not only assess what translation technologies can do, but also for whom, under what conditions, at what points in the care process, and with what short- and long-term consequences for communication, care quality, and equity in mental health. While this study focused on technological solutions, future research should be aware of intersectionality to explore how language barriers interact with broader structural inequities, such as socio-economic marginalisation, legal exclusion, and discrimination, in shaping access to care.

### Limitations

This study has several limitations. Recruitment via purposive and snowball sampling may have introduced selection bias, as individuals particularly interested in multilingualism were more likely to participate.

Although interview guides and preparatory workshops were coordinated across country teams, local adaptations and differences in interview style may have influenced the data. These differences were addressed through the development of a shared codebook and regular analytic discussions to ensure consistency.

Interviews were conducted in multiple languages and later translated into English, which may have led to the loss of cultural or linguistic nuance despite standardised procedures and the involvement of bilingual researchers. Some interviews were conducted directly in English; however, differences in participants’ first and second language varieties and proficiency levels may still have influenced how their intended meanings were conveyed.

Different country teams carried out the initial extraction of technology-related content. Given the scale and decentralised nature of the dataset, some relevant material may have been inconsistently extracted, even with shared protocols in place.

Due to the qualitative nature of the research and the fact that many participants had limited experience with translation technologies, some responses reflected expectations and hypothetical reasoning rather than extensive firsthand use. While valuable for understanding perceived barriers and needs, this limitation restricts conclusions about real-world usability.

Frequent reference to “Google Translate”, as a commonly recognised example, during interviews may have shaped participant responses and led to conflation rather than distinction of different technology types. Given the complexity of these findings, we decided not to pursue this level of differentiation in the present work, as it was beyond the scope and feasibility of this publication and warrants further investigation in future research.

Variations in sub-sample composition (e.g., Germany *n* = 46, China *n* = 23) and contextual factors may have influenced theme prominence, despite efforts to address this through collaborative coding and cross-country comparisons. These differences should be considered when interpreting cross-country comparisons, though they do not undermine the study’s aim of providing rich, contextualised insights rather than statistical generalisability.

As with all qualitative analysis, the identification and framing of themes relied on the researcher’s interpretation. Although collaborative coding, regular cross-checks, and an evolving codebook enhanced consistency, some degree of subjectivity is inherent to thematic analysis. Importantly, the aim of this study was not statistical generalisability, but rather to provide rich, contextualised insights into the use of translation technologies in mental healthcare. These insights can inform and justify future research, including larger-scale or quantitative studies, that may further explore the transferability of the findings.

While our multinational and interdisciplinary team brought diverse linguistic and cultural perspectives to the analysis, we acknowledge that our disciplinary backgrounds, linguistic positioning, and cultural backgrounds may have introduced interpretive biases. To mitigate this, we conducted reflexive discussions and involved local project partners in the theme development.

## Conclusions

This study investigated the experiences, attitudes, needs, and suggestions regarding the use of technological tools to overcome language barriers in mental healthcare, drawing on the perspectives of service users, healthcare providers, interpreters, supporters, and stakeholders across five countries. While participants acknowledged the potential of these tools to support basic communication, particularly in emergency or resource-limited settings, they also identified limitations, including issues of translation accuracy, cultural and linguistic adaptability, emotional nuance, ethical risks, and infrastructural or institutional barriers.

Most themes and sub-themes emerged across all countries and participant groups, though their salience and emphasis varied. These differences may reflect distinct policy environments, healthcare systems, and sociolinguistic contexts.

Importantly, participants did not view translation tools as stand-alone solutions but stressed embedding them in broader, system-level approaches that address the relational and emotional dimensions of mental healthcare communication. The tools’ role was primarily seen as supplementary, with a clear preference for hybrid or human-supported interpretation in complex or sensitive situations.

Equitable implementation requires that the burden of overcoming language barriers not be borne solely by individual service users or healthcare providers. Responsibility must be shared among healthcare institutions, policymakers, and technology developers to ensure that translation tools are accessible, inclusive, ethically sound, and effectively integrated into clinical workflows. Investments in infrastructure, practical training, regulatory safeguards, and participatory design processes will be essential to ensure that these technologies support, rather than undermine, safe, culturally responsive, and person-centred mental healthcare.

## Supplementary Information

Below is the link to the electronic supplementary material.

Supplementary Material 1

Supplementary Material 2

## Data Availability

No datasets were generated or analysed during the current study.

## Abbreviations

App: Application
COREQ: Consolidated criteria for reporting qualitative research
GDPR: General Data Protection Regulation
HCP: Healthcare Personnel
IP: Interpreters
SH: Stakeholder
SU: Service User
SUP: Supporter
