# Supplementary material for: Exploring the use of translation technologies to overcome language barriers in mental healthcare: a qualitative cross-country study
Source: Int J Equity Health. 2026 Mar 20;25:88. doi: 10.1186/s12939-026-02821-2 (PMC13064012; doi:10.1186/s12939-026-02821-2)
Supplement: Supplementary file 2 — Supplementary Material 2 [file 12939_2026_2821_MOESM2_ESM.pdf]

Additional file 2: Binary presence of main/sub-themes in a cross-country and cross-participant comparison.

Main themes	Sub-themes	China	Germany	The Netherlands	Romania	South Africa
Perceived effectiveness and limitations of technological tools	Limited effectiveness	All	All	All	All	All
	Context-specific suitability	All	All	All	All	HCP, SH, SU, SUP
Cultural, linguistic, and social fit	Cultural and contextual suitability	All	All	All	All	All
	Digital literacy and generational divide	HCP, IP, SU, SUP	All	SU, SUP	All	HCP, SH, SU
Human aspects of communication and care	Empathy and emotional connection	HCP, IP, SU	HCP, IP, SUP	HCP, IP, SUP	IP, SH, SU	All
	Trust and relational dynamics	HCP, IP, SU, SUP	All	HCP, IP, SH, SUP	All	HCP, IP, SU, SUP
Ethical and attitudinal dimensions	User-based reflections and perspectives	SH, SU, SUP	All	HCP, IP, SU, SUP	All	All
	Ethical considerations	All	All	HCP, SH, SU	HCP	HCP
Implementation and improvement pathways	Technical improvements	All	All	HCP, SU, SUP	All	HCP, IP, SH, SU
	Integration into existing care and workflows	IP, SH,SU, SUP	All	HCP, IP, SH, SUP	All	All
	Policy, training, and institutional support	All	All	HCP, SU	All	HCP, SH, SUP
Access barriers	Infrastructure	HCP, IP, SH, SU	All	HCP, SH, SU, SUP	All	All
	Practical constraints to tool use	IP, SH, SU	all	all	HCP, IP, SH, SU	all

HCP=Health Care Personnel, SU= Service User, SUP=Supporter, IP=Interpreter, SH=Stakeholder; All= theme present in all participant groups.
